# Allostatic load, personality traits, and cancer risk: A prospective cohort study

**DOI:** 10.1017/S0033291725102924

**Published:** 2026-01-05

**Authors:** Peng Wang, Yu Peng, Han Du, Chao Sheng, Fangfang Song, Hongji Dai, Kexin Chen

**Affiliations:** Department of Epidemiology and Biostatistics, Key Laboratory of Prevention and Control of Human Major Diseases of the Ministry of Education, Key Laboratory of Molecular Cancer Epidemiology of Tianjin, National Clinical Research Center for Cancer, Tianjin’s Clinical Research Center for Cancer, Tianjin Medical University Cancer Institute and Hospital, Tianjin Medical University, Tianjin, China

**Keywords:** allostatic Load, cancer risk, chronic stress, cohort study, personality

## Abstract

**Background:**

Cumulative stress exposure is extensively involved in carcinogenesis. However, cancer risk associated with allostatic load (AL), a valid measure of chronic stress, has not been comprehensively evaluated in large cohorts, and the combined effect of AL and personality trait on cancer risk remains unknown.

**Methods:**

This prospective cohort study was conducted based on 245,683 participants from the UK Biobank, with a median follow-up of 13.5 years. The AL score was calculated based on 11 biomarkers. Personality traits were constructed and categorized into two clusters. Multivariable Cox regression model was used to assess the risk of incident cancer according to AL and personality clusters, and multiplicative and additive interactions were evaluated.

**Results:**

High AL was associated with an increased cancer risk compared to low AL (hazard ratio [HR] = 1.06, 95% confidence interval [CI]: 1.04–1.09), particularly for cancers of stomach, liver, kidney, esophageal, lung, colorectal, breast, and leukemia (HR ranged from 1.08 to 1.43). Personality clusters was associated with risk of lung cancer (HR = 1.14, 95% CI: 1.05–1.23), but not overall cancer. Significant synergistic interaction was observed between high AL and ‘nervous-dominant’ personality for overall cancer risk, with the strongest association observed for liver cancer (HR = 1.58, 95% CI: 1.24–2.02).

**Conclusions:**

High AL was related to higher risks of overall cancer and site-specific cancers, particularly when combined with nervous-dominant personality, highlighting the interplay between chronic physiological stress and psychological factors in cancer development.

## Introduction

Cancer remains a significant public health challenge, with an estimated 20 million incident cases worldwide in 2022 and a projected 35 million cases by 2050 (Bray et al., 2024). Preclinical studies show that chronic stress can substantially promote tumorigenesis and cancer progression through multiple mechanisms, such as activation of the hypothalamic–pituitary–adrenal (HPA) axis and the sympathetic nervous system (SNS) (Cui et al., 2021), systemic inflammation (Liu, Lei, & Bai, 2025), immunosuppression (Liu et al., 2022), and epigenetic changes (Argentieri, Nagarajan, Seddighzadeh, Baccarelli, & Shields, 2017). However, population studies have yielded inconsistent results due to variability in stressor type and duration, interindividual differences in stress coping, and the subjective nature of stress exposure assessment (Ishibe, Ellison, Rao, & Lam, 2025), highlighting the value of applying objective biomarkers to evaluate lifetime stress exposure.

Allostatic load (AL), a composite metric integrating biomarkers across multiple physiological systems, offers a holistic approach to quantifying the cumulative physiological wear and tear associated with chronic stress exposure (McEwen & Stellar, 1993). AL provides a unique framework to quantify and understand the health-related effects of stress through the utilization of biomarkers reflective of the physiological dysregulation resulting from chronic exposure to stress (Barrett et al., 2022). Previous studies have demonstrated a consistent robust association between perceived stress and AL (Goldman, Glei, Seplaki, Liu, & Weinstein, 2005; Mauss & Jarczok, 2021; Morley et al., 2025), supporting AL may serve as an indicator of chronic stress exposure and reflect the physiological consequences of perceived psychological stress. While studies with limited samples have explored the associations between elevated AL and cancer mortality (Moore, Andrzejak, Bevel, Jones, & Tingen, 2022; Obeng-Gyasi et al., 2023), its role in cancer etiology remains poorly understood. A cohort study conducted in a female population reported a significant association between higher AL and overall cancer risk (Shen, Fuemmeler, Guan, & Zhao, 2022). In addition, three cohort studies have demonstrated that high AL is associated with increased risk of breast (Guan et al., 2023), lung (Guan, Shen, Zhang, Fuemmeler, & Zhao, 2024), and colorectal cancers (Zhao et al., 2025). However, there remains a lack of comprehensive analyses focusing on the relationship between AL and the incidence of overall cancer as well as a broad range of site-specific cancers among the whole population.

Furthermore, individuals show substantial variability in stress responses, with personality traits influencing physiological stress reactivity (Xin et al., 2017). Evidence indicates that higher levels of extraversion, conscientiousness, and openness, and lower levels of neuroticism, are related to less stressor-related negative affect (Leger, Charles, Turiano, & Almeida, 2016). Nevertheless, a significant gap persists in understanding whether and how individual differences in personality traits modify the association between AL and cancer risk. To fill this gap, interaction effects of AL and personality traits on cancer risk were evaluated using measures of multiplicative and additive interaction (VanderWeele & Knol, 2014). Multiplicative interaction assesses whether the joint effect of two exposures differs from the product of their individual effects on a relative risk scale, and is typically evaluated using product terms in regression models. It is most appropriate when the objective is to test statistical heterogeneity or to examine whether the relative effect of one exposure varies across levels of another. In contrast, additive interaction examines whether the combined effect of two exposures differs from the sum of their independent effects, thereby reflecting potential biological or public health interaction on an absolute risk scale. Additive interaction is particularly informative when quantifying how much of the disease risk among individuals exposed to both factors is attributable to their interaction, or when identifying subgroups in which the absolute burden of disease is greatest. Relative excess risk due to interaction (RERI) and the attributable proportion due to interaction (AP) were used to assess additive interaction. RERI quantifies the excess risk from joint exposure beyond the sum of individual risks, and AP represents the proportion of total risk attributable to the interaction itself. A RERI and AP value greater than 0 indicates synergistic interaction, whereas negative value reflects antagonistic interaction. It is generally recommended to report both multiplicative and additive interaction measures when assessing interaction (VanderWeele & Knol, 2014).

This prospective cohort study uses UK Biobank data to examine the association between AL and incident cancer risk, as well as the potential moderating effect of personality traits. The study aims to explore the complex interplay between chronic stress, personality traits, and cancer development.

## Methods

### Study population

This prospective cohort study was conducted using data from the UK Biobank, which enrolled about 500,000 participants aged 37–73 years at baseline from 22 different assessment centers across England, Scotland, and Wales between 2006 and 2010. The assessment visits comprised interviews and questionnaires covering demographic characteristics, lifestyles, physical measurements, medical information, and biological samples. The UK Biobank was approved by the National Health Service North West Multicenter Research Ethics Committee. All participants provided written informed consent.

**Figure 1.**
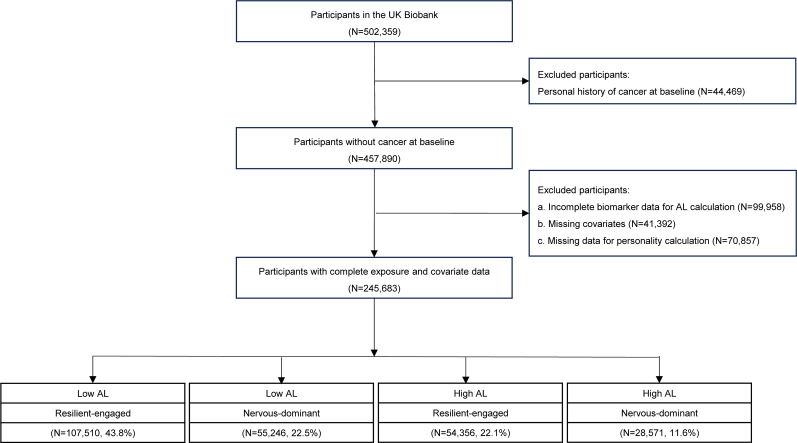
Flowchart of the study population. *Note*: AL, allostatic load.

We excluded participants with a personal history of cancer at baseline, incomplete biomarker data for AL calculation, missing covariates, and missing data in personality measurement. Finally, 245,683 participants were included in the present study (Figure 1).

### Assessment of AL

The biomarkers used to construct AL score were selected based on previous literature (Ishibe et al., 2025; Mathew et al., 2021) and UK Biobank variable availability, covering four physiologic systems: (1) metabolic: total cholesterol (TC), high-density lipoprotein cholesterol (HDL), low-density lipoprotein cholesterol (LDL), triglycerides (TG), glycated hemoglobin (HbA1c), waist-to-hip ratio (WHR); (2) cardiovascular: systolic blood pressure (SBP), diastolic blood pressure (DBP), resting pulse rate (PR); (3) inflammatory/immune biomarkers: C-reactive protein (CRP); and (4) renal: creatinine (Cre). For each biomarker (excluding HDL), a score of 1 was assigned if the measurement fell within the highest quartile (≥75th percentile). For HDL, a score of 1 was assigned for values within the lowest quartile (≤25th percentile). Otherwise, a score of 0 was assigned. A composite AL score was thus computed for each participant, ranging from 0 to 11. All participants were categorized into two groups based on their AL scores: low AL (score ≤ 3) and high AL (score > 3).

### Construction and categorization of personality traits

Using baseline touchscreen questionnaire data on psychological factors, mental health and social support, five personality dimensions – warmth, diligence, nervousness, sociability, and curiosity – were calculated using specific questionnaire items identified as proxies for the Big Five personality traits (agreeableness, conscientiousness, neuroticism, extraversion, and openness), proposed by a validated personality scale (the Big Five Inventory [BFI]) (Digman, 1990). The selected questions along with their corresponding field identifiers for all five personality traits were presented in Supplementary Table 1. Each dimension was scored by summing binary responses to related items, yielding continuous trait scores. These personality trait proxies were proposed in a study focusing on personality and myocardial infarction based on the UK Biobank (Dahlén, Miguet, Schiöth, & Rukh, 2022). Another study showed almost all phenotypic correlations among the personality trait proxies in UKB were consistent with those among the standard BFI personality traits in Human Connectome Project, supporting the reliability of personality trait proxies (Zhang et al., 2023).

Personality traits are multidimensional, and particular traits tend to co-occur or not. Given the potential intercorrelations among these proxy traits, the clustering approach allowed us to reduce dimensionality and capture participants’ overall personality patterns rather than treating each proxy as an isolated measure (Asendorpf, Borkenau, Ostendorf, & Van Aken, 2001; Donnellan & Robins, 2010). This holistic, person-centered approach may better reflect the within-individual organization of personality and the complex interplay of traits influencing health and behavior. Additionally, in the interaction analysis, grouping participants into personality clusters reduced the number of categorical levels, facilitating more parsimonious and robust models, and making it easier to identify the core interaction between AL and personality patterns on cancer risk while avoiding overfitting or instability due to sparse data. Therefore, participants were further categorized into personality clusters using K-means clustering based on the five personality traits proxies. The optimal number of clusters was determined using the elbow method.

### Assessment of outcome

The primary outcome was overall cancer incidence. The incidences of 24 site-specific cancers were additionally examined as exploratory analyses (Supplementary Table 2). Cancer cases were identified through hospital inpatient record, national cancer register data, national death register data, and self-reported data. Follow-up time was calculated from baseline recruitment until the first cancer diagnosis, death, loss to follow-up or the end of follow-up (inpatient record up to May 31, 2022, August 31, 2022, and October 31, 2022 for Wales, Scotland, and England, respectively; death records up to November 30, 2022), whichever came first.

### Covariates

Demographic variables comprised age, sex, ethnicity, and Townsend Deprivation Index. Lifestyle variables included smoking, alcohol drinking, sedentary time, regular physical activity, and healthy diet. The sedentary time was calculated as the total time spent watching TV, using a computer and driving per day. Regular physical activity was defined as engaging in ≥150 minutes of moderate activity per week or ≥75 minutes of vigorous activity per week (or an equivalent combination) (Lloyd-Jones et al., 2010). A healthy diet was defined as meeting at least four of the following criteria: Fruits: ≥ 3 servings/day; Vegetables: ≥ 3 servings/day; Fish: ≥2 servings/week; Processed meats: ≤1 serving/week; Unprocessed red meats: ≤1.5 servings/week; Whole grains: ≥3 servings/day; and Refined grains: ≤1.5 servings/day (Lourida et al., 2019). Family history of cancer was defined as having a first-degree relative diagnosed with prostate cancer, breast cancer, colorectal cancer, or lung cancer. The above covariates were collected via the touchscreen questionnaire at baseline. Body mass index (BMI) was calculated as weight in kilograms divided by the square of height in meters (kg/m^2^) during the baseline physical assessments.

### Statistical analyses

Continuous variables were summarized as mean (standard deviation) for normally distributed data and as median (interquartile range [IQR]) for non-normally distributed data, while categorical variables were presented as frequencies and percentages. Hazard ratios (HRs) and 95% confidence intervals (CIs) of cancer incidence associated with AL and clusters of personality traits were calculated using multivariable Cox proportional risk models: model 1 adjusted for age and sex; model 2 additionally adjusted for ethnicity, BMI, Townsend deprivation index, family history of cancer, smoking, alcohol drinking, sedentary time, physical activity, and healthy diet. Schoenfeld residual plots indicated no violation of proportional risk assumptions. The association between AL and cancer incidence was also assessed stratified by personality clusters, with multiplicative interactions examined by including interaction terms in the models. The joint association of AL and personality was evaluated by categorizing participants into four groups: low AL and resilient-engaged personality (reference group), low AL and nervous-dominant personality, high AL and resilient-engaged personality, and high AL and nervous-dominant personality. Additive interaction was evaluated using RERI and AP. The effect modification by sex on the association of AL and personality with cancer incidence was further evaluated. To minimize potential reverse causation, a sensitivity analysis was conducted by excluding cancer cases diagnosed within 2 years of follow-up and repeating the primary analyses. Additionally, to exam potential selection bias, we compared the demographic and lifestyle characteristics of participants included in the study and those excluded due to missing data on AL or personalities measures. We did not conduct the correction for analyses on site-specific cancers, which were considered exploratory, and the results should be interpreted with caution.

All statistical analyses were performed using SAS, version 9.4 (SAS Institute Inc., SAS Institute Inc., US) and R, version 4.5.0 (R Foundation for Statistical Computing, Vienna, Austria). All tests were two-sided, and *P* < 0.05 were considered statistically significant.

## Results

### Baseline characteristics of study population

**Figure 2.**
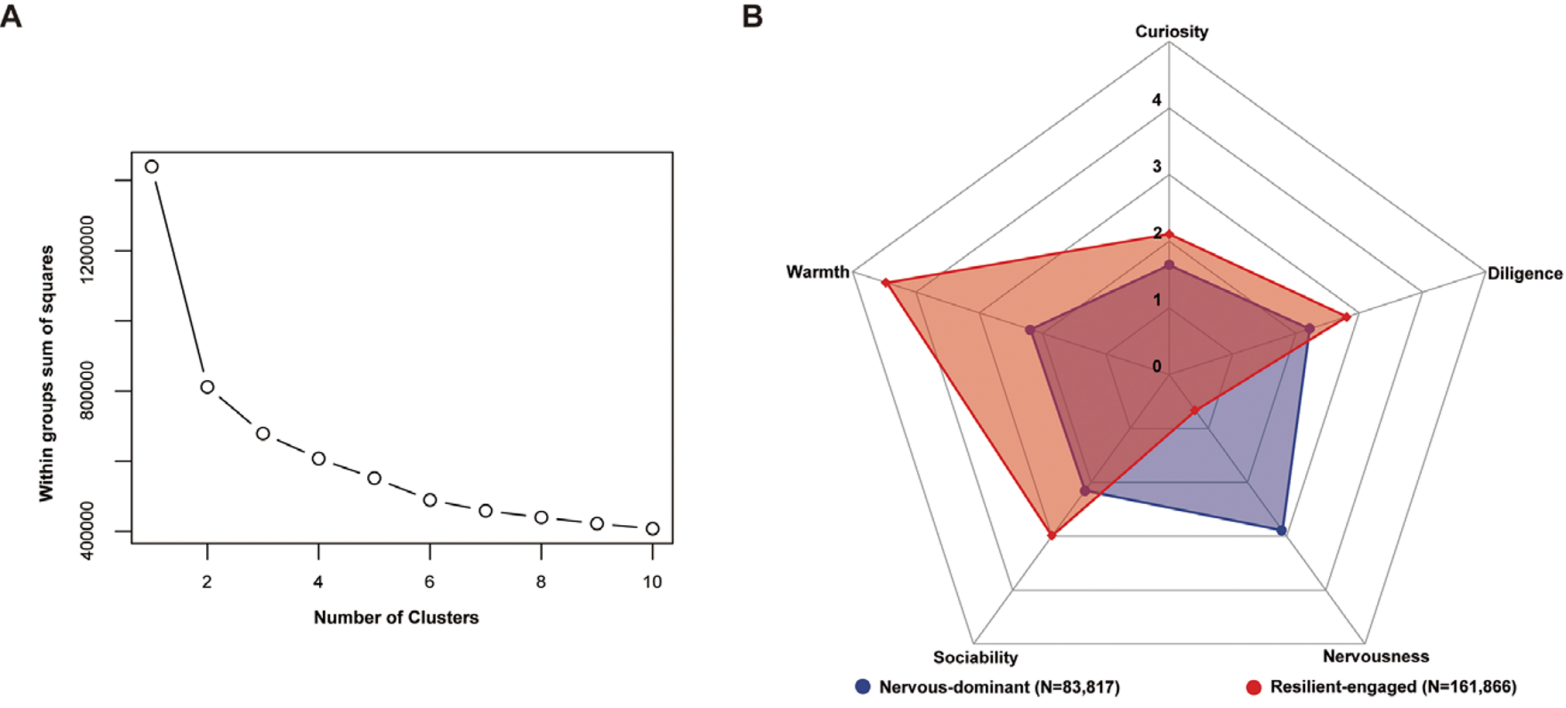
Characterization of personality clusters derived from K-means clustering. (A) Scree plot indicating the optimal cluster number. (B) Radar chart visualizing the identified personality clusters across five personality components.

Two mutually exclusive personality clusters were identified using the elbow method (Figure 2A). Cluster 1 was labeled ‘resilient-engaged’ due to higher scores of warmth, sociability, diligence, and curiosity, but a lower nervousness score (N = 161,866). In contrast, Cluster 2 had a higher level of nervousness but lower scores on the other four personality dimensions and was thus termed ‘nervous-dominant’ (N = 83,817) (Figure 2B).

Participants were categorized into four groups based on AL and personality clusters: low AL and ‘resilient-engaged’ personality (N = 107,510, 43.8%), low AL and ‘nervous-dominant’ personality (N = 55,246, 22.5%), high AL and ‘resilient-engaged’ personality (N = 54,356, 22.1%), and high AL and ‘nervous-dominant’ personality (N = 28,571, 11.6%). Compared with those characterized by low AL and ‘resilient-engaged’ personality, participants with low AL and ‘nervous-dominant’ personality were more likely to be younger, female, non-White, have higher Townsend Deprivation Index and a family history of cancer, and engage in unhealthy behaviors such as smoking, physical inactivity, sedentary lifestyle, and unhealthy diet. While participants with high AL and ‘resilient-engaged’ or ‘nervous-dominant’ personality appeared to be older, male, non-White, have a higher Townsend Deprivation Index, with a family history of cancer, showing more unfavorable lifestyle, including obesity, smoking, reduced physical activity, prolonged sedentary time, and a less healthy diet (Table 1). Additionally, baseline characteristics were largely comparable between participants included in the study and those excluded due to missing data on AL or personality measures (Supplementary Table 3).

**Table 1.** Baseline characteristics of study population by AL and personality

Characteristics	Low AL		High AL	
	Resilient-engaged	Nervous-dominant	Resilient-engaged	Nervous-dominant
	(N = 107,510, 43.8%)	(N = 55,246, 22.5%)	(N = 54,356, 22.1%)	(N = 28,571, 11.6%)
**Age, years, median (IQR)**	57 (49, 62)	54 (47, 61)	60 (53, 64)	58 (51, 63)
**Sex, n (%)**				
Female	63,835 (59.4)	35,170 (63.7)	17,927 (33.0)	10,092 (35.3)
Male	43,675 (40.6)	20,076 (36.3)	36,429 (67.0)	18,479 (64.7)
**Ethnicity, n (%)**				
White	103,895 (96.6)	53,118 (96.2)	52,410 (96.4)	27,388 (95.9)
Asian	1,414 (1.3)	827 (1.5)	843 (1.6)	572 (2.0)
Black	1,063 (1.0)	567 (1.0)	576 (1.1)	274 (1.0)
Mixed	533 (0.5)	349 (0.6)	230 (0.4)	125 (0.4)
Other	605 (0.6)	385 (0.7)	297 (0.5)	212 (0.7)
**BMI, kg/m** ^**2**^**, n (%)**				
<18.5	600 (0.6)	477 (0.9)	50 (0.1)	26 (0.1)
18.5–24.9	46,104 (42.9)	23,634 (42.8)	7,540 (13.9)	3,662 (12.8)
25–29.9	45,638 (42.5)	22,180 (40.2)	25,758 (47.4)	12,414 (43.5)
≥30	15,168 (14.0)	8,955 (16.1)	21,008 (38.6)	12,469 (43.6)
**Townsend deprivation index, n (%)**				
<−3.64	30,366 (28.2)	13,949 (25.3)	14,498 (26.7)	6,593 (23.1)
−3.64 to −2.14	28,496 (26.5)	13,788 (25.0)	14,441 (26.6)	6,895 (24.1)
−2.14 to 0.54	27,443 (25.5)	13,937 (25.2)	13,767 (25.3)	7,072 (24.8)
≥0.55	21,205 (19.8)	13,572 (24.5)	11,650 (21.4)	8,011 (28.0)
**Smoking, n (%)**				
Never	64,843 (60.3)	30,077 (54.4)	28,197 (51.9)	13,084 (45.8)
Previous	34,582 (32.2)	19,107 (34.6)	20,292 (37.3)	11,179 (39.1)
Current	8,085 (7.5)	6,062 (11.0)	5,867 (10.8)	4,308 (15.1)
**Alcohol drinking, n (%)**				
Never	3,631 (3.4)	1,780 (3.2)	1,992 (3.7)	1,101 (3.9)
Previous	2,721 (2.5)	2,041 (3.7)	1,697 (3.1)	1,482 (5.2)
Current	101,158 (94.1)	51,425 (93.1)	50,667 (93.2)	25,988 (90.9)
**Physical activity, n (%)**				
No	43,069 (40.1)	24,877 (45.0)	25,765 (47.4)	15,070 (52.8)
Yes	64,441 (59.9)	30,369 (55.0)	28,591 (52.6)	13,501 (47.2)
**Sedentary time, h/day, n (%)**				
<4	43,618 (40.6)	20,249 (36.7)	14,328 (26.4)	6,391 (22.4)
4–6	47,624 (44.3)	24,551 (44.4)	26,185 (48.2)	13,325 (46.6)
>6	16,268 (15.1)	10,446 (18.9)	13,843 (25.4)	8,855 (31.0)
**Healthy diet, n (%)**				
No	49,588 (46.1)	28,049 (50.8)	32,197 (59.2)	18,285 (64.0)
Yes	57,922 (53.9)	27,197 (49.2)	22,159 (40.8)	10,286 (36.0)
**Family history of cancer, n (%)**				
No	69,738 (64.9)	35,577 (64.4)	34,551 (63.6)	17,987 (63.0)
Yes	37,772 (35.1)	19,669 (35.6)	19,805 (36.4)	10,584 (37.0)

*Note*: AL, allostatic load; BMI, body mass index; IQR, interquartile range.

### Association between AL and cancer risk

**Figure 3.**
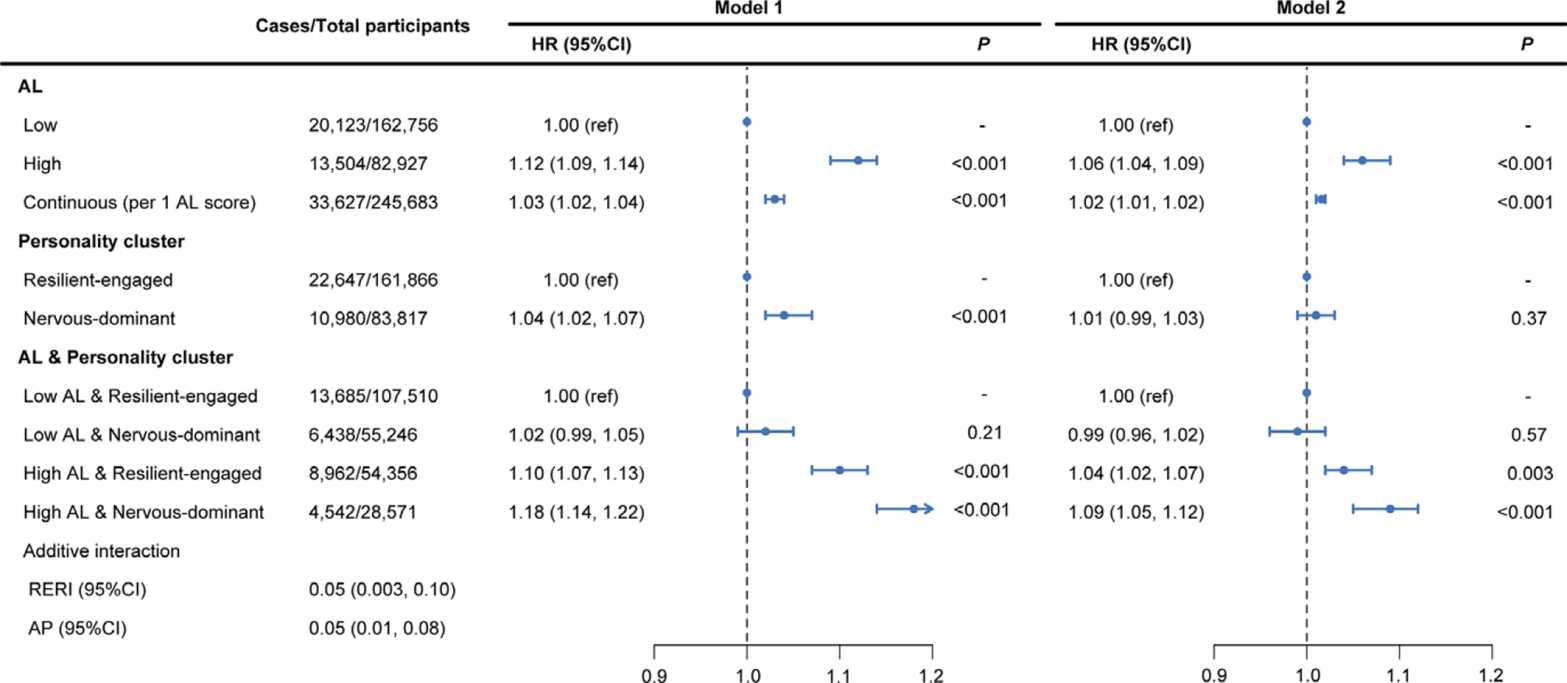
Risk of incident cancer according to AL and personality clusters. Model 1: adjusted for age, sex. Model 2: further adjusted for ethnicity, BMI, Townsend deprivation index, family history of cancer, smoking, alcohol drinking, sedentary time, physical activity and healthy diet. *Note*: AL, allostatic load; AP, attributable proportion due to interaction; BMI, body mass index; CI, confidence interval; HR, hazard ratio; RERI, relative excess risk due to interaction.

During a median follow-up period of 13.5 years, 33,627 incident cancer cases were recorded. In the fully adjusted model, compared to participants with low AL, those with high AL exhibited an elevated risk of overall cancer (HR = 1.06, 95% CI: 1.04–1.09, Figure 3). Each 1-point increase in AL was associated with a 2% higher risk of overall cancer (Figure 3). Similar associations of AL and overall cancer risk were observed after excluding cancer cases occurred within 2 years of follow-up (Supplementary Table 4). For site-specific cancers, AL was positively associated with stomach cancer (HR = 1.43, 95% CI: 1.21–1.69); liver cancer (HR = 1.37, 95% CI: 1.14–1.64); kidney cancer (HR = 1.34, 95% CI: 1.17–1.53); esophageal cancer (HR = 1.29, 95% CI: 1.11–1.49); lung cancer (HR = 1.19, 95% CI: 1.10–1.30); colorectal cancer (HR = 1.17, 95% CI: 1.09–1.25); leukemia (HR = 1.15, 95% CI: 1.00–1.31); and breast cancer (HR = 1.08, 95% CI: 1.01–1.15) in the fully adjusted model (Supplementary Table 5).

### Personality clusters and cancer risk

We observed a significant association between ‘nervous-dominant’ personality and overall cancer risk (HR = 1.04, 95% CI: 1.02–1.07) in the age- and sex-adjusted model, while this association was not statistically significant in the fully adjusted models (HR = 1.01, 95% CI: 0.99–1.03) (Figure 3). Similar results were observed after excluding cancer cases occurred within 2 years of follow-up (Supplementary Table 4). When considering different cancer sites, in the age- and sex-adjusted model, ‘nervous-dominant’ personality was linked to increased risks of lung cancer (HR = 1.41, 95% CI: 1.30–1.52); esophageal cancer (HR = 1.23, 95% CI: 1.07–1.42); and liver cancer (HR = 1.23, 95% CI: 1.04–1.47) compared with ‘resilient-engaged’ personality; however, in fully adjusted models, a significant association was observed only for lung cancer (HR = 1.14, 95% CI: 1.05–1.23) (Supplementary Table 6).

### Association of AL with cancer risk according to personality clusters

The association between AL and overall cancer risk differed significantly across personality clusters (Supplementary Table 7). We observed a more pronounced increase in overall cancer risk related to high AL among participants with ‘nervous-dominant’ personality (HR = 1.10, 95% CI: 1.06–1.15) than those with ‘resilient-engaged’ personality (HR = 1.04, 95% CI: 1.01–1.07) in the fully adjusted model, demonstrating a significant multiplicative interaction (*P* for interaction = 0.04) (Supplementary Table 7). Similar multiplicative interaction was observed in the sensitivity analysis (Supplementary Table 4). Additionally, significant multiplicative interactions were also found for several site-specific cancers, including liver, oral, mesothelioma, testicular, ovarian, and endometrial cancers. Specifically, compared to those with ‘resilient-engaged’ personality, individuals with ‘nervous-dominant’ personality exhibited a more pronounced increased risk associated with high AL for liver, oral, mesothelioma, testicular, and ovarian cancers. Conversely, for endometrial cancer, the elevated risk associated with high AL was greater among those with ‘resilient-engaged’ personality (Supplementary Table 8).

### Joint associations of AL and personality with cancer risk

Significant additive interaction was observed between high AL and ‘nervous-dominant’ personality for overall cancer risk (RERI = 0.05, 95% CI: 0.003–0.10; AP = 0.05, 95% CI: 0.01–0.08) (Figure 3). Compared to participants with low AL and a ‘resilient-engaged’ personality, those with high AL and ‘nervous-dominant’ personality had the highest overall cancer risk (HR = 1.09, 95% CI: 1.05–1.12). Results were consistent in the sensitivity analysis (Supplementary Table 4). For site-specific cancers, the synergistic effect of high AL and ‘nervous-dominant’ personality was observed for liver (RERI = 0.53, 95% CI: 0.14–0.92; AP = 0.33, 95% CI: 0.10–0.57), oral (RERI = 0.30, 95% CI: 0.02–0.59; AP = 0.30, 95% CI: 0.03–0.57), mesothelioma (RERI = 0.54, 95% CI: 0.07–0.12; AP = 0.47, 95% CI: 0.005–0.94), testicular (RERI = 1.25, 95% CI: 0.21–2.30; AP = 0.74, 95% CI: −0.50–1.99), and ovarian cancers (RERI = 0.32, 95% CI: 0.02–0.61; AP = 0.34, 95% CI: 0.04–0.63). Among these, the risk of liver cancer associated with high AL and ‘nervous-dominant’ personality was the highest (HR = 1.58, 95% CI: 1.24–2.02). Notably, antagonistic effects of high AL and ‘nervous-dominant’ personality on endometrial cancer risk was observed (RERI = -0.42, 95% CI: −0.79, −0.09; AP = -0.45, 95% CI: −0.68, −0.22) (Supplementary Table 9).

### Subgroup analysis

**Figure 4.**
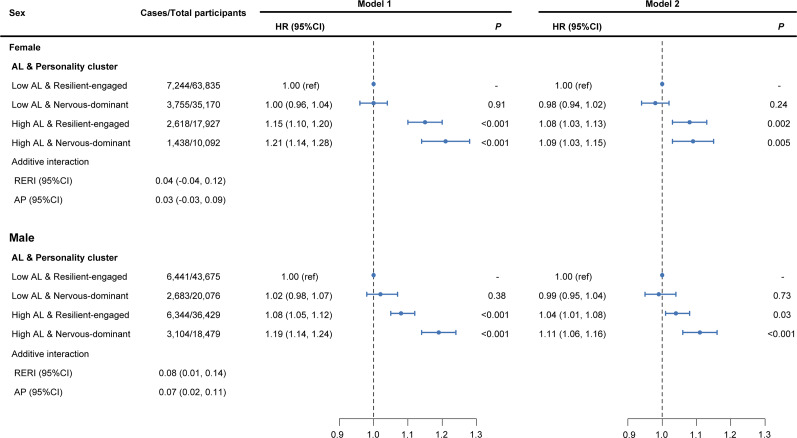
Risk of incident cancer according to AL and personality clusters stratified by sex. Model 1: adjusted for age. Model 2: further adjusted for ethnicity, BMI, Townsend deprivation index, family history of cancer, smoking, alcohol drinking, sedentary time, physical activity and healthy diet. *Note*: AL, allostatic load; AP, attributable proportion due to interaction; BMI, body mass index; CI, confidence interval; HR, hazard ratio; RERI, relative excess risk due to interaction.

We also examined the association of AL and personality with cancer risk separately in male and female groups (Figure 4; Supplementary Table 10). Compared with low AL and ‘resilient-engaged’ personality, high AL and ‘nervous-dominant’ personality consistently associated with the highest overall cancer risk in both males (HR = 1.11, 95% CI: 1.06–1.16) and females (HR = 1.09, 95% CI: 1.03–1.15) in the fully adjusted model. Notably, the additive interactions between high AL and ‘nervous-dominant’ personality on overall cancer risk were only observed in males (RERI = 0.08, 95% CI: 0.01–0.14; AP = 0.07, 95% CI: 0.02–0.11) (Figure 4). Similarly, we observed that the elevated overall cancer risk associated with high AL was greater among those with ‘nervous-dominant’ personality than ‘resilient-engaged’ personality in both males and females, yet a significant multiplicative interaction was found only in males (*P* for interaction = 0.03) (Supplementary Table 10).

## Discussion

In this large-scale prospective cohort study, we found that high AL was significantly associated with a higher risk of overall cancer and multiple site-specific cancers, such as cancers of the stomach, liver, kidney, esophageal, lung, colorectal, breast, and leukemia. The ‘nervous-dominant’ personality appeared to amplify the cancer risk associated with high AL. The joint effect of AL and personality traits was also observed for liver, oral, mesothelioma, testicular, and ovarian cancer.

Supportive evidence indicates that AL may serve as a good measure of chronic stress, capturing the intersectionality between external stressors and multisystem physiological response to them (Ishibe et al., 2025). However, the existing evidence linking AL to cancer risk predominantly derives from cross-sectional designs (Mattei, Demissie, Falcon, Ordovas, & Tucker, 2010; Parente, Hale, & Palermo, 2012) or focuses on limited cancer sites (Guan et al., 2023, 2024; Zhao et al., 2025). A cohort study based on 3015 midlife females demonstrated that high AL was linked to increased overall cancer risk (Shen et al., 2022). Consistently, our study found a positive association between AL and risk of overall cancer based on a large-scale cohort covering both sexes, with stronger effects observed in males compared to females. For site-specific cancers, besides the reported breast (Guan et al., 2023), lung (Guan et al., 2024), and colorectal cancer (Zhao et al., 2025), we also found a positive association between AL and the risk of stomach, liver, kidney, esophageal cancer, and leukemia. These findings provide evidence from population studies for the hypothesis that chronic stress may serve as a shared upstream determinant in cancer etiology across multiple sites.

Preclinical evidence indicates that chronic stress fuels malignant progression by remodeling core hallmarks of cancer (Cui et al., 2021). Stress stimulates neuroendocrine system, consisting of the HPA axis and the SNS, resulting in abnormal release of hormones. Persistent elevations in cortisol and catecholamines promote tumorigenesis through directly targeting cancer cells (Yan et al., 2023) and indirectly affecting the tumor immune microenvironment mechanisms (Cui et al., 2021). For stomach cancer, studies have shown that catecholamines upregulate MMP-7 expression through the β2-AR-mediated signaling pathway, thereby driving cancer invasion and metastasis (Christodoulidis, Konstantinos-Eleftherios, & Marina-Nektaria, 2024; Shi et al., 2010). Besides, recent study from NHANES demonstrated that higher AL scores were significantly associated with hepatic steatosis and fibrosis (Dai & Zhou, 2024), established precursors to hepatocellular carcinoma, implying inflammatory and metabolic dysregulations may represent the mechanistic bridge between chronic stress and hepatocarcinogenesis. The mechanisms underlying the association between AL and kidney cancer are not clear; however, key components of the AL, such as hypertension, obesity, and related metabolic syndrome, and inflammation, are independent risk factors for kidney cancer (Campi et al., 2023), which may explain the observed association. Additionally, a study uncovered that chronic stress-induced persistent glucocorticoid elevation stimulates cholesterol uptake, triggering dysregulated cholesterol metabolism that significantly promotes esophageal carcinogenesis (Wang et al., 2025). Another study revealed that chronic stress promotes the progression of human pre-B-cell acute lymphoblastic leukemia in orthotopic mouse models through indirect pathways regulated by β-adrenergic signaling (Lamkin et al., 2012), emphasizing the role of neuroendocrine pathways in the stress-cancer link.

Moreover, our study suggests that the ‘nervous-dominant’ personality traits may exacerbate the carcinogenic effects of AL. A meta-analysis study indicated significant positive association between neuroticism and AL, but inverse associations of conscientiousness, openness with AL (Yoneda et al., 2023). The ‘nervous-dominant’ personality cluster – marked by anxiety, depression, and a propensity toward negative and instable emotionality, may represent a phenotype with heightened vulnerability to stressors, reduced coping capacity, greater physiological reactivity, and poorer health behavior, amplifying chronic stress effects (Yoneda et al., 2023). In contrast, individuals with high openness may handle potentially stressful situations with greater flexibility and adaptability (Penley & Tomaka, 2002), and individuals with high conscientiousness tend to engage in healthier behaviors, leading to better physiological regulation (Milad & Bogg, 2020), which may attenuate the adverse effects of stress on cancer. Similar to the pattern observed for overall cancer risk, AL and the ‘nervous-dominant’ personality exhibited synergistic effects for most site-specific cancers (liver, oral, mesothelioma, testicular, and ovarian cancers), although antagonistic effects for certain cancers such as endometrial cancer. Individuals with nervous-dominant personality tend to be more sensitive to stress, exhibiting hyperactivation of the HPA axis and enhanced cortisol responses (Mangold & Wand, 2006), which may suppress hypothalamic–pituitary–gonadal axis driven estrogen synthesis (Nielsen et al., 2007), thereby antagonizing the effects of stress-induced metabolic and immune dysregulation on endometrial cancer risk. Nevertheless, this interpretation should be viewed with caution, and warrants further investigation. For liver cancer, the observed synergistic effect between high AL and a ‘nervous-dominant’ personality may reflect the potential compounding effect of psychosocial and physiological stressors on liver cancer development. Chronic stress may activate the HPA axis and promote systemic inflammation and immune dysregulation (Liu et al., 2025), both of which have been implicated in hepatocarcinogenesis (Brouillet & Lafdil, 2023; Yahoo, Dudek, Knolle, & Heikenwalder, 2023). Additionally, nervous-dominant individuals may adopt unhealthy behaviors such as poor diet, alcohol use, and inactivity, further increasing liver cancer risk (Cheraghpour, Hatami, & Singal, 2025). These findings underscore the need to consider both personality traits and biological stress markers in liver cancer prevention strategies.

In addition, the observed sex differences, in which joint effects were more pronounced in males than females, may reflect sex differences in stress reactivity and coping behaviors. This was supported by existing literature suggesting that male may have greater physiological reactivity to stress (Liu & Zhang, 2020) and were less likely to engage in protective behaviors such as help-seeking or emotional disclosure (Taylor et al., 2000). These differences may partially explain the stronger synergistic effect observed between AL and ‘nervous-dominant’ personality in males.

Our findings highlight the value of incorporating AL and personality profiling into precision cancer prevention strategies. Interventions targeting stress reduction (Eckerling, Ricon-Becker, Sorski, Sandbank, & Ben-Eliyahu, 2021) and enhancement of adaptive personality traits (Olaru et al., 2023) may yield meaningful cancer risk reductions, particularly among psychologically vulnerable individuals. Public health screening tools that assess both AL and personality contribute to early identification of high-risk individuals.

## Strengths and Limitations

The major strengths of this study include its prospective cohort design, large sample size, long follow-up period (median 13.5 years), comprehensive assessment of risk profiles for 24 site-specific cancers associated with AL, as well as systematic interaction analyses to evaluate effect modifications by personality traits. However, some limitations exist. First, the study population was predominantly White. The lack of diversity in population limited the generalization of the findings to other populations. Second, AL was assessed based on objective biomarkers, without incorporating the comprehensive clinimetric evaluation (Fava et al., 2019). As a result, biomarker-derived AL may misclassify individuals, underestimating those with high psychosocial stress but without overt physiological dysregulation, and potentially overestimating those with low stress but aberrant biomarker levels. This nondifferential exposure misclassification likely attenuated the observed associations. Third, the baseline assessment of AL may not capture its temporal dynamics during follow-up. The lack of repeated AL measurements could contribute to random measurement error and attenuate true associations over time. Fourth, personality traits were not measured via standardized BFI instruments. Although UK Biobank questionnaires provide reasonable proxies of BFI, the results should be interpreted with caution, and further validation of these proxy measures is warranted. Fifth, the elbow method used to determine the optimal number of personality clusters may yield subjective results and obscure complex or overlapping subgroup structures. Sixth, given the nature of observational studies, residual confounding may still exist, even after adjusting for a range of confounders.

## Conclusions

In summary, our findings suggest that elevated AL was significantly associated with an increased cancer risk, particularly when combined with a ‘nervous-dominant’ personality, highlighting the interplay between chronic physiological stress and psychological factors in carcinogenesis. Future research is warranted to elucidate the underlying biological mechanisms and to explore whether targeted interventions aimed at reducing AL can mitigate cancer risk, while incorporating individual differences in personality traits.

## Supporting information

10.1017/S0033291725102924.sm001 Wang et al. supplementary material Wang et al. supplementary material

## Data Availability

The data can be requested through application to the UK Biobank (https://www.ukbiobank.ac.uk/).

## Abbreviations

AL: allostatic load
AP: attributable proportion due to interaction
BFI: Big Five Inventory
BMI: body mass index
CI: confidence interval
Cre: creatinine
CRP: C-reactive protein
DBP: diastolic blood pressure
HbA1c: glycated hemoglobin
HDL: high-density lipoprotein cholesterol
HPA: hypothalamic–pituitary–adrenal
HR: hazard ratio
ICD: International Classification of Diseases
IQR: interquartile range
LDL: low-density lipoprotein cholesterol
PR: resting pulse rate
RERI: relative excess risk due to interaction
SBP: systolic blood pressure
SMD: standardized mean difference
SNS: sympathetic nervous system
TC: total cholesterol
TG: triglycerides
TME: tumor immune microenvironment
WHR: waist-to-hip ratio

## Supplementary material

The supplementary material for this article can be found at http://doi.org/10.1017/S0033291725102924.
