# Supplementary material for: Allostatic load, personality traits, and cancer risk: A prospective cohort study
Source: Psychol Med. 2026 Jan 5;56:e7. doi: 10.1017/S0033291725102924 (PMC13095360; doi:10.1017/S0033291725102924)
Supplement: Wang et al. supplementary material [file S0033291725102924sup001.docx]

**Allostatic load, personality traits, and cancer Risk: A prospective cohort study**

**Supplementary Table 1.** **The codes for defining personality traits.**

**Supplementary Table 2.** **The codes for defining cancers.**

**Supplementary Table 3. Baseline characteristics of participants included in the study and participants excluded due to missing data for AL and personalities measure.**

**Supplementary Table 4. Risk of incident cancer according to AL and personality clusters after exclusion of cancer cases occurred within 2 years of follow-up.**

**Supplementary Table 5. Risk of incident site-specific cancers associated with high AL compared with low AL.**

**Supplementary Table 6. Risk of incident site-specific cancers associated with nervous-dominant personality compared with resilient-engaged personality.**

**Supplementary Table 7. Risk of incident overall cancers associated with high AL stratified by personality clusters.**

**Supplementary Table 8. Risk of incident site-specific cancers associated with high AL stratified by personality clusters.**

**Supplementary Table 9. Risk of incident site-specific cancers according to AL and personality clusters.**

**Supplementary Table 10. Risk of incident overall cancers associated with high AL stratified by personality clusters and sex.**

**Supplementary Table 1. The codes for defining personality traits.**

| **Personality traits** | **Questionnaire items** | **Field** |
| --- | --- | --- |
| Warmth (Agreeableness) | Able to confide to (> once a month) | 2110 |
|  | Irritability (no) | 1940 |
|  | Mood swings (no) | 1920 |
|  | Tense / 'highly strung' (no) | 1990 |
|  | Nervous feelings (no) | 1970 |
| Diligence  (Conscientiousness) | Frequency of enthusiasm/disinterest in last 2 weeks (not at all) | 2060 |
|  | Fed-up feelings (no) | 1960 |
|  | Risk taking (no) | 2040 |
|  | Worry too long after embarrassment (yes) | 2000 |
| Nervousness  (Neuroticism) | Tense / 'highly strung' (yes) | 1990 |
|  | Irritability (yes) | 1940 |
|  | Frequency of enthusiasm/disinterest in last 2 weeks (> several days) | 2060 |
|  | Mood swings (yes) | 1920 |
|  | Sensitivity/hurt feelings (yes) | 1950 |
| Sociability  (Extraversion) | Frequency of friend/family visit (> once a month) | 1031 |
|  | Guilty feelings (no) | 2030 |
|  | Frequency of tiredness/lethargy in last 2 weeks (not at all) | 2080 |
|  | Leisure/social activities | 6160 |
| Curiosity  (Openness) | Loneliness, isolation (no) | 2020 |
|  | Suffer from 'nerves' (no) | 2010 |
|  | Frequency of tenseness/restlessness in last 2 weeks (> several days) | 2070 |
|  | Risk taking (yes) | 2040 |

**Supplementary Table 2.** **The codes for defining cancers.**

|  | **Self-reported cancer codes (Data-Field 20001)** | **ICD-10 codes**  **(Data-Field 41270,40001,40002,40006)** | **ICD-9 codes**  **(Data-Field 41271,40013)** |
| --- | --- | --- | --- |
| Overall cancer | 1001-1088 (except 1060-1062, 1073) | C00-C97 (except C44) | 140-208 (except 173) |
| Oral | 1004,1005,1010,1011,1012, 1015,1016,1077,1078,1079 | C00-C14 | 140-149 |
| Esophageal | 1017 | C15 | 150 |
| Stomach | 1018 | C16 | 151 |
| Liver | 1024 | C22 | 155 |
| Pancreatic | 1026, 1088 | C25 | 157 |
| Colorectal | 1020,1022,1023,1086 | C18-C20 | 153-154 |
| Lung | 1001,1027,1028 | C34 | 162 |
| Kidney | 1034 | C64-C65 | 1890-1891 |
| Bladder | 1035 | C67 | 188 |
| Thyroid | 1065 | C73 | 193 |
| Bone | 1063,1085 | C40-C41 | 170 |
| Brain | 1032 | C71 | 191 |
| Malignant melanoma | 1059 | C43 | 172 |
| Non-Hodgkin lymphoma | 1053 | C82-C85 | 202 |
| Hodgkin lymphoma | 1052 | C81 | 201 |
| Multiple myeloma | 1050 | C90 | 203 |
| Leukemia | 1048 | C91-C95 | 204-208 |
| Mesothelioma | 1064 | C45 | - |
| Prostate | 1044 | C61 | 185 |
| Testicular | 1045 | C62 | 186 |
| Breast | 1002 | C50 | 174 |
| Cervical | 1041,1072 | C53 | 180 |
| Endometrial | 1040 | C54.1 | 182 |
| Ovarian | 1039 | C56 | 1830 |

**Supplementary Table 3. Baseline characteristics of participants included in the study and participants excluded due to missing data for AL and personalities measure.**

|  | **Participants included in the study (N=245,683)** | **Participants excluded due to missing data for AL and personalities measure (N=153,853)** | **Standardized mean difference (SMD)** |
| --- | --- | --- | --- |
| **Age, years, median (IQR)** | 57 (49,63) | 57 (50,63) | 0.04 |
| **Sex, n (%)** |  |  |  |
| Female | 127,024 (51.7) | 86,231 (56.1) | 0.09 |
| Male | 118,659 (48.3) | 67,622 (44.0) |  |
| **Ethnicity, n (%)** |  |  |  |
| White | 236,811 (96.4) | 143,627 (93.4) | 0.10 |
| Asian | 3,656 (1.5) | 4,455 (2.9) | 0.03 |
| Black | 2,480 (1.0) | 3,099 (2.0) | 0.04 |
| Mixed | 1,237 (0.5) | 960 (0.6) | 0.01 |
| Other | 1,499 (0.6) | 1,712 (1.1) | 0.02 |
| **BMI, kg/m^2^, n (%)** |  |  |  |
| <18.5 | 1,153 (0.5) | 857 (0.6) | 0.01 |
| 18.5-24.9 | 80,940 (32.9) | 51,440 (33.4) | 0.01 |
| 25-29.9 | 105,990 (43.1) | 64,748 (42.1) | 0.02 |
| ≥30 | 57,600 (23.4) | 36,808 (23.9) | 0.01 |
| **Townsend deprivation index, n (%)** |  |  |  |
| <-3.64 | 65,406 (26.6) | 38,191 (24.8) | 0.04 |
| -3.64--2.14 | 63,620 (25.9) | 38,213 (24.8) | 0.02 |
| -2.14-0.54 | 62,219 (25.3) | 38,661 (25.1) | 0.005 |
| ≥0.55 | 54,438 (22.2) | 38,788 (25.2) | 0.07 |
| **Smoking, n (%)** |  |  |  |
| Never | 136,201 (55.4) | 85,737 (55.7) | 0.01 |
| Previous | 85,160 (34.7) | 52,348 (34.0) | 0.01 |
| Current | 24,322 (9.9) | 15,768 (10.3) | 0.01 |
| **Alcohol drinking, n (%)** |  |  |  |
| Never | 8,504 (3.5) | 7,144 (4.6) | 0.06 |
| Previous | 7,941 (3.2) | 5,236 (3.4) | 0.01 |
| Current | 229,238 (93.3) | 141,473 (92.0) | 0.05 |
| **Physical activity, n (%)** |  |  |  |
| No | 108,781 (44.3) | 72,318 (47.0) | 0.05 |
| Yes | 136,902 (55.7) | 81,535 (53.0) |  |
| **Sedentary time, h/day, n (%)** |  |  |  |
| <4 | 84,586 (34.4) | 53,223 (34.6) | 0.003 |
| 4-6 | 111,685 (45.5) | 69,339 (45.1) | 0.01 |
| >6 | 49,412 (20.1) | 31,291 (20.3) | 0.01 |
| **Healthy diet, n (%)** |  |  |  |
| No | 128,119 (52.2) | 81,654 (53.1) | 0.02 |
| Yes | 117,564 (47.8) | 72,199 (46.9) |  |
| **Family history of cancer, n (%)** |  |  |  |
| No | 157,853 (64.3) | 99,511 (64.7) | 0.01 |
| Yes | 87,830 (35.8) | 54,342 (35.3) |  |

The standardized mean difference (SMD) was used to compared the difference between groups, with an SMD greater than 0.1 indicating meaningful difference.

Abbreviations: AL, allostatic load; BMI, body mass index; IQR, interquartile range; SMD, Standardized Mean Difference.

**Supplementary Table 4. Risk of incident cancer according to AL and personality clusters after exclusion of cancer cases occurred within 2 years of follow-up.**

|  | **Cases/Total participants** | **Model 1** | | **Model 2** | |
| --- | --- | --- | --- | --- | --- |
|  |  | **HR (95%CI)** | ***P*** | **HR (95%CI)** | ***P*** |
| **AL** |  |  |  |  |  |
| Low | 17,950/160,341 | 1.00 (ref) | - | 1.00 (ref) | - |
| High | 11,978/81,145 | 1.11 (1.08, 1.14) | <0.001 | 1.05 (1.03, 1.08) | <0.001 |
| Continuous (per 1 AL score) | 29,928/241,486 | 1.03 (1.02, 1.04) | <0.001 | 1.01 (1.00, 1.02) | <0.001 |
| **Personality cluster** |  |  |  |  |  |
| Resilient-engaged | 20,149/159,087 | 1.00 (ref) | - | 1.00 (ref) | - |
| Nervous-dominant | 9,779/82,399 | 1.04 (1.02, 1.07) | <0.001 | 1.01 (0.99, 1.04) | 0.41 |
| **AL & Personality cluster** |  |  |  |  |  |
| Low AL & Resilient-engaged | 12,227/105,922 | 1.00 (ref) | - | 1.00 (ref) | - |
| Low AL & Nervous-dominant | 5,723/54,419 | 1.01 (0.98, 1.05) | 0.42 | 0.99 (0.95, 1.02) | 0.35 |
| High AL & Resilient-engaged | 7,922/53,165 | 1.09 (1.05, 1.12) | <0.001 | 1.03 (1.00, 1.06) | 0.04 |
| High AL & Nervous-dominant | 4,056/27,980 | 1.18 (1.13, 1.22) | <0.001 | 1.08 (1.04, 1.12) | <0.001 |
| P for trend |  | <0.001 |  | <0.001 |  |
| P for multiplicative interaction | 0.01 | | | | |
| Additive interaction |  |  |  |  |  |
| RERI (95%CI) | 0.07 (0.02, 0.12) | | | | |
| AP (95%CI) | 0.06 (0.02, 0.10) | | | | |

Model 1: adjusted for age, sex.

Model 2: further adjusted for ethnicity, BMI, Townsend deprivation index, family history of cancer, smoking, alcohol drinking, sedentary time, physical activity and healthy diet.

Abbreviations: AL, allostatic load; AP, attributable proportion due to interaction; BMI, body mass index; CI, confidence interval; HR, hazard ratio; RERI, relative excess risk due to interaction.

**Supplementary Table 5. Risk of incident site-specific cancers associated with high AL compared with low AL.**

| **Cancer site** | **High AL (N=82,927)** | | | | |
| --- | --- | --- | --- | --- | --- |
|  | **Cases (%)** | **Model 1** | | **Model 2** | |
|  |  | **HR (95%CI)** | ***P*** | **HR (95%CI)** | ***P*** |
| Oral | 284(0.3) | 1.03 (0.88, 1.20) | 0.74 | 1.01 (0.85, 1.19) | 0.95 |
| Esophageal | 449(0.5) | 1.58 (1.37, 1.81) | <0.001 | 1.29 (1.11, 1.49) | <0.001 |
| Stomach | 359(0.4) | 1.74 (1.45, 2.03) | <0.001 | 1.43 (1.21, 1.69) | <0.001 |
| Liver | 301(0.4) | 1.63 (1.38, 1.94) | <0.001 | 1.37 (1.14, 1.64) | <0.001 |
| Pancreatic | 446(0.5) | 1.28 (1.12, 1.45) | <0.001 | 1.11 (0.97, 1.27) | 0.13 |
| Colorectal | 1,738(2.1) | 1.24 (1.16, 1.32) | <0.001 | 1.17 (1.09, 1.25) | <0.001 |
| Lung | 1,253(1.5) | 1.41 (1.30, 1.53) | <0.001 | 1.19 (1.10, 1.30) | <0.001 |
| Kidney | 550(0.7) | 1.56 (1.38, 1.77) | <0.001 | 1.34 (1.17, 1.53) | <0.001 |
| Bladder | 830(1.0) | 1.22 (1.11, 1.35) | <0.001 | 1.08 (0.98, 1.20) | 0.14 |
| Thyroid | 99(0.1) | 1.36 (1.05, 1.76) | 0.02 | 1.23 (0.94, 1.62) | 0.13 |
| Bone | 31(0.04) | 0.72 (0.45, 1.12) | 0.15 | 0.72 (0.45, 1.16) | 0.18 |
| Brain | 223(0.3) | 1.09 (0.92, 1.30) | 0.34 | 1.08 (0.90, 1.30) | 0.42 |
| Malignant melanoma | 778(0.9) | 0.97 (0.89, 1.06) | 0.53 | 1.01 (0.92, 1.11) | 0.88 |
| Non-Hodgkin lymphoma | 558(0.7) | 0.95 (0.85, 1.06) | 0.38 | 0.92 (0.82, 1.03) | 0.15 |
| Hodgkin lymphoma | 46(0.1) | 1.05 (0.72, 1.54) | 0.79 | 0.86 (0.58, 1.29) | 0.47 |
| Multiple myeloma | 251(0.3) | 0.91 (0.77, 1.07) | 0.24 | 0.87 (0.74, 1.03) | 0.11 |
| Leukemia | 458(0.6) | 1.20 (1.05, 1.36) | 0.006 | 1.15 (1.00, 1.31) | 0.04 |
| Mesothelioma | 122(0.2) | 0.99 (0.78, 1.27) | 0.95 | 1.00 (0.77, 1.29) | 0.99 |
| Prostate | 3,560(6.5) | 0.93 (0.89, 0.97) | <0.001 | 0.97 (0.92, 1.02) | 0.19 |
| Testicular | 28(0.1) | 0.99 (0.60, 1.61) | 0.95 | 1.03 (0.60, 1.77) | 0.92 |
| Breast | 1,445(5.2) | 1.11 (1.05, 1.18) | <0.001 | 1.08 (1.01, 1.15) | 0.02 |
| Cervical | 33(0.1) | 1.37 (0.91, 2.06) | 0.13 | 1.24 (0.81, 1.91) | 0.32 |
| Endometrial | 308(1.1) | 1.55 (1.34, 1.78) | <0.001 | 1.15 (0.99, 1.33) | 0.06 |
| Ovarian | 199(0.7) | 1.04 (0.89, 1.23) | 0.61 | 0.96 (0.81, 1.14) | 0.61 |

Model 1: adjusted for age, sex.

Model 2: further adjusted for ethnicity, BMI, Townsend deprivation index, family history of cancer, smoking, alcohol drinking, sedentary time, physical activity and healthy diet. Female reproductive factors including menarche age, age at first live birth, parity, hormone replacement therapy, oral contraceptive pill and menopause state were additionally adjusted in the models of breast cancer, cervical cancer, endometrial cancer and ovarian cancer.

The low AL was used as the reference group (N=162756).

The analysis for prostate cancer and testicular cancer was conducted among males, while the analysis for breast cancer, cervical cancer, endometrial cancer and ovarian cancer was conducted among females.

The numbers of males in low AL and high AL were 63751 and 54908, respectively.

The numbers of females in low AL and high AL were 99005 and 28019, respectively.

Abbreviations: AL, allostatic load; BMI, body mass index; CI, confidence interval; HR, hazard ratio.

**Supplementary Table 6. Risk of incident site-specific cancers associated with nervous-dominant personality compared with resilient-engaged personality.**

| **Cancer site** | **Nervous-dominant (N=83,817)** | | | | |
| --- | --- | --- | --- | --- | --- |
|  | **Cases (%)** | **Model 1** | | **Model 2** | |
|  |  | **HR (95%CI)** | ***P*** | **HR (95%CI)** | ***P*** |
| Oral | 232 (0.3) | 0.99 (0.84, 1.16) | 0.88 | 0.91 (0.77, 1.06) | 0.23 |
| Esophageal | 289 (0.3) | 1.23 (1.07, 1.42) | 0.005 | 1.10 (0.95, 1.27) | 0.20 |
| Stomach | 214 (0.3) | 1.11 (0.94, 1.31) | 0.22 | 1.02 (0.86, 1.20) | 0.82 |
| Liver | 198 (0.2) | 1.23 (1.04, 1.47) | 0.02 | 1.11 (0.93, 1.32) | 0.26 |
| Pancreatic | 323 (0.4) | 1.10 (0.96, 1.26) | 0.17 | 1.04 (0.91, 1.20) | 0.53 |
| Colorectal | 1,297 (1.6) | 1.06 (0.99, 1.13) | 0.09 | 1.03 (0.96, 1.10) | 0.42 |
| Lung | 1,017 (1.2) | 1.41 (1.30, 1.52) | <0.001 | 1.14 (1.05, 1.23) | 0.001 |
| Kidney | 341 (0.4) | 1.05 (0.92, 1.20) | 0.44 | 0.99 (0.87, 1.13) | 0.93 |
| Bladder | 514 (0.6) | 1.00 (0.90, 1.11) | 0.94 | 0.92 (0.83, 1.03) | 0.14 |
| Thyroid | 104 (0.1) | 1.10 (0.86, 1.40) | 0.46 | 1.06 (0.83, 1.36) | 0.64 |
| Bone | 32 (0.04) | 1.17 (0.76, 1.80) | 0.48 | 1.13 (0.73, 1.75) | 0.57 |
| Brain | 171 (0.2) | 0.94 (0.78, 1.13) | 0.49 | 0.93 (0.77, 1.11) | 0.41 |
| Malignant melanoma | 646 (0.8) | 0.88 (0.80, 0.96) | 0.005 | 0.91 (0.83, 1.00) | 0.06 |
| Non-Hodgkin lymphoma | 487 (0.6) | 1.08 (0.97, 1.21) | 0.16 | 1.08 (0.97, 1.21) | 0.17 |
| Hodgkin lymphoma | 42 (0.1) | 1.11 (0.77, 1.62) | 0.58 | 1.09 (0.74, 1.59) | 0.67 |
| Multiple myeloma | 210 (0.3) | 1.01 (0.86, 1.19) | 0.92 | 1.01 (0.86, 1.20) | 0.87 |
| Leukemia | 343 (0.4) | 1.12 (1.98, 1.27) | 0.10 | 1.09 (0.95, 1.24) | 0.21 |
| Mesothelioma | 79 (0.1) | 1.04 (0.80, 1.35) | 0.77 | 1.01 (0.78, 1.32) | 0.94 |
| Prostate | 2,265 (5.9) | 1.00 (0.95, 1.05) | 0.87 | 1.01 (0.96, 1.06) | 0.73 |
| Testicular | 28 (0.1) | 1.37 (0.84, 2.24) | 0.21 | 1.43 (0.87, 2.35) | 0.15 |
| Breast | 2,043 (4.5) | 0.98 (0.93, 1.04) | 0.47 | 0.97 (0.92, 1.02) | 0.25 |
| Cervical | 44 (0.1) | 0.92 (0.64, 1.34) | 0.67 | 0.89 (0.62, 1.30) | 0.55 |
| Endometrial | 306 (0.7) | 0.95 (0.83, 1.09) | 0.48 | 0.94 (0.82, 1.08) | 0.40 |
| Ovarian | 243 (0.5) | 0.86 (0.74, 0.99) | 0.04 | 0.83 (0.72, 0.97) | 0.02 |

Model 1: adjusted for age, sex.

Model 2: further adjusted for ethnicity, BMI, Townsend deprivation index, family history of cancer, smoking, alcohol drinking, sedentary time, physical activity and healthy diet. Female reproductive factors including menarche age, age at first live birth, parity, hormone replacement therapy, oral contraceptive pill and menopause state were additionally adjusted in the models of breast cancer, cervical cancer, endometrial cancer and ovarian cancer.

The resilient-engaged personality was used as the reference group (N=161,866).

The analysis for prostate cancer and testicular cancer was conducted among males, while the analysis for breast cancer, cervical cancer, endometrial cancer and ovarian cancer was conducted among females.

The numbers of males in low AL and high AL were 80,104 and 38,555, respectively.

The numbers of females in low AL and high AL were 81,762 and 45,262, respectively.

Abbreviations: BMI, body mass index; CI, confidence interval; HR, hazard ratio.

**Supplementary Table 7. Risk of incident overall cancers associated with high AL stratified by personality clusters.**

|  | **Cases/Total participants** | **Model 1** | | | | **Model 2** | | | **P for interaction** |
| --- | --- | --- | --- | --- | --- | --- | --- | --- | --- |
|  |  | **HR (95%CI)** | | ***P*** | | **HR (95%CI)** | | ***P*** |  |
| Resilient-engaged |  |  |  | |  | |  | | 0.04 |
| Low AL | 13,685/107,510 | 1.00 (ref) | - | | 1.00 (ref) | | - | |  |
| High AL | 8,962/54,356 | 1.10 (1.07, 1.13) | <0.001 | | 1.04 (1.01, 1.07) | | 0.01 | |  |
| Nervous-dominant |  |  |  | |  | |  | |  |
| Low AL | 6,438/55,246 | 1.00 (ref) | - | | 1.00 (ref) | | - | |  |
| High AL | 4,542/28,571 | 1.16 (1.12, 1.21) | <0.001 | | 1.10 (1.06, 1.15) | | <0.001 | |  |

Model 1: adjusted for age, sex.

Model 2: further adjusted for ethnicity, BMI, Townsend deprivation index, family history of cancer, smoking, alcohol drinking, sedentary time, physical activity and healthy diet.

Abbreviations: AL, allostatic load; BMI, body mass index; CI, confidence interval; HR, hazard ratio.

**Supplementary Table 8. Risk of incident site-specific cancers associated with high AL stratified by** **personality clusters.**

| **Cancer site** | **Resilient-engaged** | | **Nervous-dominant** | | ***P* for interaction** |
| --- | --- | --- | --- | --- | --- |
|  | **High AL** | | **High AL** | |  |
|  | **HR (95%CI)** | ***P*** | **HR (95%CI)** | ***P*** |  |
| Oral | 0.93 (0.76, 1.14) | 0.48 | 1.18 (0.88, 1.57) | 0.27 | 0.04 |
| Esophageal | 1.20 (1.00, 1.44) | 0.05 | 1.48 (1.15, 1.92) | 0.003 | 0.35 |
| Stomach | 1.45 (1.18, 1.77) | <0.001 | 1.40 (1.04, 1.88) | 0.03 | 0.62 |
| Liver | 1.17 (0.94, 1.47) | 0.16 | 1.83 (1.34, 2.51) | <0.001 | 0.02 |
| Pancreatic | 1.02 (0.86, 1.20) | 0.85 | 1.34 (1.05, 1.70) | 0.02 | 0.10 |
| Colorectal | 1.15 (1.05, 1.24) | 0.002 | 1.22 (1.08, 1.38) | 0.001 | 0.36 |
| Lung | 1.14 (1.02, 1.26) | 0.02 | 1.28 (1.12, 1.47) | <0.001 | 0.17 |
| Kidney | 1.35 (1.15, 1.58) | <0.001 | 1.33 (1.05, 1.68) | 0.02 | 0.37 |
| Bladder | 1.06 (0.94, 1.20) | 0.33 | 1.13 (0.93, 1.36) | 0.22 | 0.61 |
| Thyroid | 1.30 (0.93, 1.84) | 0.13 | 1.12 (0.71, 1.77) | 0.62 | 0.60 |
| Bone | 0.58 (0.33, 1.04) | 0.07 | 1.11 (0.51, 2.45) | 0.79 | 0.64 |
| Brain | 1.03 (0.82, 1.29) | 0.83 | 1.22 (0.87, 1.70) | 0.25 | 0.16 |
| Malignant melanoma | 0.97 (0.86, 1.09) | 0.56 | 1.11 (0.93, 1.33) | 0.24 | 0.20 |
| Non-Hodgkin lymphoma | 0.86 (0.75, 0.99) | 0.04 | 1.05 (0.86, 1.28) | 0.64 | 0.13 |
| Hodgkin lymphoma | 0.95 (0.58, 1.56) | 0.83 | 0.72 (0.37, 1.41) | 0.33 | 0.71 |
| Multiple myeloma | 0.85 (0.70, 1.05) | 0.13 | 0.92 (0.67, 1.25) | 0.58 | 0.95 |
| Leukemia | 1.16 (0.98, 1.37) | 0.08 | 1.13 (0.89, 1.43) | 0.32 | 0.63 |
| Mesothelioma | 0.83 (0.61, 1.13) | 0.23 | 1.57 (0.96, 2.55) | 0.07 | 0.03 |
| Prostate | 0.95 (0.89, 1.00) | 0.05 | 1.03 (0.94, 1.12) | 0.59 | 0.11 |
| Testicular | 0.73 (0.34, 1.57) | 0.42 | 1.52 (0.67, 3.48) | 0.32 | 0.02 |
| Breast | 1.07 (0.99, 1.16) | 0.09 | 1.09 (0.98, 1.22) | 0.12 | 0.78 |
| Cervical | 1.27 (0.73, 2.19) | 0.39 | 1.21 (0.60, 2.44) | 0.59 | 0.55 |
| Endometrial | 1.32 (1.11, 1.57) | 0.002 | 0.86 (0.66, 1.12) | 0.25 | 0.01 |
| Ovarian | 0.89 (0.72, 1.09) | 0.26 | 1.13 (0.84, 1.52) | 0.42 | 0.04 |

Models were adjusted for age, sex, ethnicity, BMI, Townsend deprivation index, family history of cancer, smoking, alcohol drinking, sedentary time, physical activity and healthy diet. Female reproductive factors including menarche age, age at first live birth, parity, hormone replacement therapy, oral contraceptive pill and menopause state were additionally adjusted in the models of breast cancer, cervical cancer, endometrial cancer and ovarian cancer.

The low AL was used as the reference group across different personality clusters.

The analysis for prostate cancer and testicular cancer was conducted among males, while the analysis for breast cancer, cervical cancer, endometrial cancer and ovarian cancer was conducted among females.

Abbreviations: AL, allostatic load; BMI, body mass index; CI, confidence interval; HR, hazard ratio.

**Supplementary Table 9.** **Risk of incident site-specific cancers according to AL and personality clusters.**

| **Cancer site** | **HR (95%CI)** | | | | **Additive interactions** | |
| --- | --- | --- | --- | --- | --- | --- |
|  | **Low AL** | **Low AL** | **High AL** | **High AL** | **RERI** | **AP** |
|  | **Resilient-engaged** | **Nervous-dominant** | **Resilient-engaged** | **Nervous-dominant** |  |  |
| Oral | 1.00 (ref) | 0.79 (0.64, 0.98) | 0.90 (0.74, 1.10) | 0.99 (0.79, 1.26) | 0.30 (0.02, 0.59) | 0.30 (0.03, 0.57) |
| Esophageal | 1.00 (ref) | 1.02 (0.82, 1.26) | 1.23 (1.03, 1.47) | 1.43 (1.17, 1.76) | 0.18 (-0.16, 0.52) | 0.13 (-0.06, 0.32) |
| Stomach | 1.00 (ref) | 1.07 (0.83, 1.36) | 1.47 (1.20, 1.79) | 1.44 (1.13, 1.83) | -0.09 (-0.51, 0.33) | -0.06 (-0.28, 0.15) |
| Liver | 1.00 (ref) | 0.88 (0.67, 1.14) | 1.18 (0.95, 1.47) | 1.58 (1.24, 2.02) | 0.53 (0.14, 0.92) | 0.33 (0.10, 0.57) |
| Pancreatic | 1.00 (ref) | 0.94 (0.78, 1.13) | 1.03 (0.88, 1.22) | 1.22 (1.00, 1.48) | 0.24 (-0.04, 0.52) | 0.20 (0.003, 0.40) |
| Colorectal | 1.00 (ref) | 1.00 (0.91, 1.09) | 1.15 (1.06, 1.24) | 1.22 (1.10, 1.35) | 0.07 (-0.07, 0.22) | 0.06 (-0.04, 0.16) |
| Lung | 1.00 (ref) | 1.08 (0.97, 1.21) | 1.14 (1.03, 1.27) | 1.38 (1.23, 1.55) | 0.16 (-0.02, 0.34) | 0.11 (0.006, 0.22) |
| Kidney | 1.00 (ref) | 1.06 (0.88, 1.27) | 1.39 (1.19, 1.62) | 1.30 (1.08, 1.58) | -0.15 (-0.46, 0.17) | -0.11 (-0.29, 0.06) |
| Bladder | 1.00 (ref) | 0.90 (0.78, 1.04) | 1.07 (0.94, 1.20) | 1.01 (0.87, 1.18) | 0.05 (-0.15, 0.25) | 0.05 (-0.11, 0.20) |
| Thyroid | 1.00 (ref) | 1.11 (0.82, 1.50) | 1.30 (0.93, 1.80) | 1.26 (0.84, 1.87) | -0.14 (-0.77, 0.49) | -0.11 (-0.49, 0.26) |
| Bone | 1.00 (ref) | 1.05 (0.62, 1.81) | 0.67 (0.38, 1.19) | 0.88 (0.45, 1.72) | 0.15 (-0.64, 0.94) | 0.17 (-0.62, 0.96) |
| Brain | 1.00 (ref) | 0.83 (0.65, 1.06) | 1.00 (0.80, 1.24) | 1.07 (0.82, 1.41) | 0.24 (-0.10, 0.59) | 0.23 (-0.06, 0.52) |
| Malignant melanoma | 1.00 (ref) | 0.87 (0.78, 0.98) | 0.97 (0.87, 1.09) | 0.96 (0.83, 1.11) | 0.11 (-0.07, 0.29) | 0.12 (-0.04, 0.27) |
| Non-Hodgkin lymphoma | 1.00 (ref) | 1.01 (0.88, 1.16) | 0.87 (0.76, 1.00) | 1.04 (0.88, 1.23) | 0.17 (-0.05, 0.39) | 0.16 (-0.01, 0.34) |
| Hodgkin lymphoma | 1.00 (ref) | 1.03 (0.64, 1.67) | 0.82 (0.51, 1.33) | 0.97 (0.55, 1.74) | 0.13 (-0.60, 0.86) | 0.13 (-0.48, 0.74) |
| Multiple myeloma | 1.00 (ref) | 1.01 (0.82, 1.25) | 0.87 (0.71, 1.06) | 0.89 (0.69, 1.15) | 0.007 (-0.31, 0.32) | 0.007 (-0.26, 0.28) |
| Leukemia | 1.00 (ref) | 1.12 (0.94, 1.33) | 1.17 (1.00, 1.37) | 1.23 (1.01, 1.50) | -0.06 (-0.36, 0.25) | -0.05 (-0.23, 0.14) |
| Mesothelioma | 1.00 (ref) | 0.76 (0.52, 1.12) | 0.84 (0.62, 1.14) | 1.14 (0.79, 1.64) | 0.54 (0.07, 1.02) | 0.47 (0.005, 0.94) |
| Prostate | 1.00 (ref) | 0.97 (0.91, 1.04) | 0.95 (0.89, 1.00) | 1.00 (0.93, 1.07) | 0.08 (-0.02, 0.17) | 0.08 (0.005, 0.15) |
| Testicular | 1.00 (ref) | 0.83 (0.41, 1.69) | 0.60 (0.29, 1.25) | 1.68 (0.87, 3.25) | 1.25 (0.21, 2.30) | 0.74 (-0.50, 1.99） |
| Breast | 1.00 (ref) | 0.97 (0.91, 1.04) | 1.08 (1.00, 1.17) | 1.04 (0.94, 1.14) | -0.03 (-0.15, 0.10) | -0.03 (-0.12, 0.07) |
| Cervical | 1.00 (ref) | 0.83 (0.54, 1.29) | 1.14 (0.67, 1.93) | 1.22 (0.65, 2.27) | 0.25 (-0.65, 1.15) | 0.20 (-0.43, 0.83) |
| Endometrial | 1.00 (ref) | 1.06 (0.90, 1.26) | 1.30 (1.09, 1.54) | 0.94 (0.74, 1.19) | -0.42 (-0.75, -0.09) | -0.45 (-0.68, -0.22) |
| Ovarian | 1.00 (ref) | 0.76 (0.63, 0.91) | 0.85 (0.69, 1.05) | 0.93 (0.72, 1.20) | 0.32 (0.02, 0.61) | 0.34 (0.04, 0.63) |

Models were adjusted for age, sex, ethnicity, BMI, Townsend deprivation index, family history of cancer, smoking, alcohol drinking, sedentary time, physical activity and healthy diet. Female reproductive factors including menarche age, age at first live birth, parity, hormone replacement therapy, oral contraceptive pill and menopause state were additionally adjusted in the models of breast cancer, cervical cancer, endometrial cancer and ovarian cancer.

The analysis for prostate cancer and testicular cancer was conducted among males, while the analysis for breast cancer, cervical cancer, endometrial cancer and ovarian cancer was conducted among females.

Abbreviations: AL, allostatic load; AP, attributable proportion due to interaction; BMI, body mass index; CI, confidence interval; HR, hazard ratio; RERI, relative excess risk due to interaction.

**Supplementary Table 10. Risk of incident overall cancers associated with high AL stratified by personality clusters and sex.**

|  | **Cases/Total participants** | **Model 1** | | | | **Model 2** | | | **P for interaction** |
| --- | --- | --- | --- | --- | --- | --- | --- | --- | --- |
|  |  | **HR (95%CI)** | | ***P*** | | **HR (95%CI)** | | ***P*** |  |
| **Female** |  |  |  | |  | |  | |  |
| Resilient-engaged |  |  |  | |  | |  | | 0.38 |
| Low AL | 7,244/63,835 | 1.00 (ref) | - | | 1.00 (ref) | | - | |  |
| High AL | 2,618/17,927 | 1.15 (1.10, 1.21) | <0.001 | | 1.08 (1.03, 1.13) | | 0.002 | |  |
| Nervous-dominant |  |  |  | |  | |  | |  |
| Low AL | 3,755/35,170 | 1.00 (ref) | - | | 1.00 (ref) | | - | |  |
| High AL | 1,438/10,092 | 1.20 (1.12, 1.27) | <0.001 | | 1.12 (1.05, 1.20) | | <0.001 | |  |
| **Male** |  |  |  | |  | |  | |  |
| Resilient-engaged |  |  |  | |  | |  | | 0.03 |
| Low AL | 6,441/43,675 | 1.00 (ref) | - | | 1.00 (ref) | | - | |  |
| High AL | 6,344/36,429 | 1.08 (1.05, 1.12) | <0.001 | | 1.04 (1.00, 1.08) | | 0.03 | |  |
| Nervous-dominant |  |  |  | |  | |  | |  |
| Low AL | 2,683/20,076 | 1.00 (ref) | - | | 1.00 (ref) | | - | |  |
| High AL | 3,104/18,479 | 1.16 (1.10, 1.22) | <0.001 | | 1.12 (1.06, 1.18) | | <0.001 | |  |

Model 1: adjusted for age.

Model 2: further adjusted for ethnicity, BMI, Townsend deprivation index, family history of cancer, smoking, alcohol drinking, sedentary time, physical activity and healthy diet.

Abbreviations: AL, allostatic load; BMI, body mass index; CI, confidence interval; HR, hazard ratio.
